# Disrupted rich club network in behavioral variant frontotemporal dementia and early‐onset Alzheimer's disease

**DOI:** 10.1002/hbm.23069

**Published:** 2015-12-17

**Authors:** Madelaine Daianu, Adam Mezher, Mario F. Mendez, Neda Jahanshad, Elvira E. Jimenez, Paul M. Thompson

**Affiliations:** ^1^ Imaging Genetics Center, Mark & Mary Stevens Neuroimaging & Informatics Institute University of Southern California Marina del Rey California; ^2^ Department of Neurology UCLA School of Medicine Los Angeles California; ^3^ Department of Neurology Behavioral Neurology Program, UCLA Los Angeles California; ^4^ Departments of Neurology, Psychiatry, Radiology, Engineering, Pediatrics, and Ophthalmology University of Southern California Los Angeles California

**Keywords:** early onset Alzheimer's disease, behavioral variant frontotemporal dementia, rich club, diffusion tensor imaging, connectome

## Abstract

In network analysis, the so‐called “rich club” describes the core areas of the brain that are more densely interconnected among themselves than expected by chance, and has been identified as a fundamental aspect of the human brain connectome. This is the first in‐depth diffusion imaging study to investigate the rich club along with other organizational changes in the brain's anatomical network in behavioral frontotemporal dementia (bvFTD), and a matched cohort with early‐onset Alzheimer's disease (EOAD). Our study sheds light on how bvFTD and EOAD affect connectivity of white matter fiber pathways in the brain, revealing differences and commonalities in the connectome among the dementias. To analyze the breakdown in connectivity, we studied three groups: 20 bvFTD, 23 EOAD, and 37 healthy elderly controls. All participants were scanned with diffusion‐weighted magnetic resonance imaging (MRI), and based on whole‐brain probabilistic tractography and cortical parcellations, we analyzed the rich club of the brain's connectivity network. This revealed distinct patterns of disruption in both forms of dementia. In the connectome, we detected less disruption overall in EOAD than in bvFTD [false discovery rate (FDR) critical *P*_perm_ = 5.7 × 10^−3^, 10,000 permutations], with more involvement of richly interconnected areas of the brain (chi‐squared *P* = 1.4 × 10^−4^)—predominantly posterior cognitive alterations. In bvFTD, we found a greater spread of disruption including the rich club (FDR critical *P*_perm_ = 6 × 10^−4^), but especially more peripheral alterations (chi‐squared *P* = 6.5 × 10^−3^), particularly in medial frontal areas of the brain, in line with the known behavioral socioemotional deficits seen in these patients. *Hum Brain Mapp 37:868–883, 2016*. © **2015 The Authors. Human Brain Mapping Published by Wiley Periodicals, Inc**.

**Abbreviations**
AD: Alzheimer's disease
bvFTD: behavioral variant frontotemporal dementia
FA: fractional anisotropy
FDR: false discovery rate
FTD: frontotemporal dementia
EOAD: early‐onset Alzheimer's disease
DMN: default mode network
DTI: diffusion tensor imaging
DWI: diffusion‐weighted imaging
*E*: number of edges in network
LOAD: late‐onset Alzheimer's disease
MD: mean diffusivity
MRI: magnetic resonance imaging
NS: not significant
*k*: nodal degree
ROI: region of interest
Φ^w^: weighted rich club
Φ_n_: normalized weighted rich club
SD: standard deviation
SN: salience network
TE: echo time
TI: inversion time
TR: relaxation time

## INTRODUCTION

The human nervous system is a network of connections that supports complex communication between numerous brain regions. Hundreds of billions of densely interconnected neurons are involved in disseminating, transforming, and processing signals through hundreds of trillions of synapses. Using emerging network‐sensitive neuroimaging techniques, such as diffusion‐weighted imaging (DWI), we are able to reconstruct the gross organization of the human brain as a structural network of connections that make up the “connectome”, revealing the architectural properties of the nervous system. Studies of the human connectome have advanced neuroscience by shedding light on the cognitive and behavioral characteristics [Toga and Thompson, 2013] of the healthy and diseased living brain.

Neurodegenerative diseases may target specific neural networks with a characteristic profile of anatomical progression [Braak and Braak, 1991; Zhou et al., 2012]. For instance, in late‐onset Alzheimer's disease (LOAD), pathology may emerge first in the more central and interconnected areas of the brain's network, known as “hubs” [Buckner et al., 2005]. Network hubs form a high‐capacity central core, or rich club, and rich club connections play a key role in global integration of information among regions of the brain [van den Heuvel and Sporns, 2011]. Many connectivity studies hypothesize either that disease may target certain connections in the rich club network [Crossley et al., 2014], or that network disruptions in the rich club have the most significant impact on cognition. One plausible hypothesis of neurodegenerative disease progression relates to “transneuronal spread”, which may involve prion‐like mechanisms [Frost and Diamond, 2010], that promote and propagate the transfer of toxic agents between the interconnected components of the connectome [Zhou et al., 2012]. In line with this, another recently proposed disease model in neurodegeneration was based on the diffusive spread of disease causing agents (i.e., *tau*, amyloid) interpreted through longitudinal structural networks. This model was found to recapitulate classic patterns of alterations in Alzheimer's disease (AD) and behavioral variant frontotemporal dementia (bvFTD) [Raj et al., 2012].

In this work, we used diffusion imaging to reconstruct the brain's structural connectome in 20 patients with bvFTD, 23 age‐matched patients with early‐onset Alzheimer's disease (EOAD) and compared both groups to 37 age‐matched cognitively healthy controls. This is the first study to combine these two unique forms of disease and analyze their network disturbances within the “rich club” framework. bvFTD and EOAD are prototypical neurodegenerative diseases comparable in age and onset that tend to affect partially distinct neuroanatomical regions and arise due to different processes [Daianu et al., 2015dd]. After AD, FTD is the second most common form of neurodegenerative dementia [Koedam et al., 2010], while bvFTD—one of the subtypes of FTD, is associated with frontal, temporal, and insular degeneration [Seeley, 2008]. bvFTD patients typically present with deficits in emotion, social conduct, and insight [Carr et al., 2015; Mendez and Shapira, 2011]. bvFTD is known to begin focally with different foci depending on the subtype, affecting neighboring regions in the brain. Meanwhile, EOAD—a relatively rare form of AD, presents with slightly different patterns of degeneration from LOAD, affecting more posterior regions of the brain, including the posterior cingulate and precuneus [Karas et al., 2007]. Prominent and presenting symptoms of EOAD involve cognitive domains, such as language and visuospatial skills [Frisoni et al., 2007; Ishii et al., 2005; Karas et al., 2007], rather than memory.

Here, we further analyzed potential global disruptions in the rich club organization of the brain in bvFTD and EOAD, in relation to the white matter integrity of the fibers linking various regions in the connectome. To obtain a comprehensive landscape of disruptions, we reconstructed our networks using information from DTI metrics fractional anisotropy and mean diffusivity, in addition to commonly studied fiber density‐based networks. From a regional approach, we defined centrally located nodes among the hubs of each network in conjunction with more peripheral areas of the brain. Moreover, we assessed the altered proportion of white matter connections leading to altered information transfer between hub and nonhub regions. We reasoned that the key hubs in the rich club would be located in the main sites of atrophy in the two diseases—predominantly in the frontal lobe in bvFTD and parietal lobe in EOAD patients. We aimed to reveal distinct patterns of disruption in the networks of bvFTD and EOAD, highlighting key nodes and connections within the rich club network, or among its peripheral regions, that may enable the spread of pathological processes leading to structural and functional consequences of brain disease.

## METHODS

### Participants and Image Preprocessing

Twenty bvFTD patients (60.7 years ± 10.7 SD), 23 age‐matched EOAD patients (59.6 years ± 8.8 SD) and 37 cognitively healthy individuals (59.4 years ± 9.6 SD) were scanned with whole‐brain structural T1‐weighted magnetic resonance imaging (MRI) and diffusion‐weighted MRI (Table 1). Participants with bvFTD and with EOAD were recruited from an outpatient behavioral neurology clinic in an academic university medical center. bvFTD participants met criteria for “probable” bvFTD based on International Consensus Criteria [Rascovsky et al., 2011] and frontotemporal changes on neuroimaging. While it is not possible to absolutely rule out atypical AD pathology among those who meet clinical criteria for probable bvFTD, these criteria have a reported specificity for frontotemporal lobar degeneration pathology close to 95% [Harris et al., 2013; Lamarre et al., 2013]. The bvFTD cohort was reasonably homogeneous with prominent apathy, episodic disinhibition, stereotypical behaviors, emotional blunting, and carbohydrate craving. The reason for the greater homogeneity relates, in part, to the absence of patients with any of the known mutations. Participants with EOAD were diagnosed according to the National Institute of Aging‐Alzheimer's criteria for clinically probable AD [McKhann et al., 2011]. Given the usual presenile onset of bvFTD, to have age‐matched groups, only EOAD patients with early‐onset disease (<65 years of age) were included in the comparison group. On presentation, most of the EOAD patients had predominant memory impairment, four had visuospatial deficits, and five had language impairment, however, they all had similar AD biomarker changes in the cerebrospinal fluid. All of the EOAD patients were nonfamilial, that is, there was no history of autosomal dominant transmission or affected family members with EOAD. All of the EOAD patients had the diagnosis of AD confirmed by the presence of low β42‐amyloid and high total *tau* and phospho‐*tau* on cerebrospinal fluid analysis. None of the EOAD patients had a prior history of a psychiatric disorder or neurological disease and none was at the time taking medications that could impact performance on the neurological exam. In addition, there were no bvFTD or EOAD patients with clinical or neuroimaging evidence of comorbid neurological disorders such as dementia with Lewy bodies. PET imaging with florbetapir or other amyloid‐binding radioligands was not available at the date of most MRI scan acquisitions. Across all three groups, individuals with major medical illnesses (except hypertension or diabetes) were excluded.

**Table 1:** Demographic information for 37 healthy controls, 20 bvFTD, and 23 EOAD patients

	Controls	bvFTD	EOAD	Total
Age	59.4 ± 9.6 SD	60.7 ± 10.7 SD	59.0 ± 5.0 SD	59.6 ± 8.8 SD
Sex	17M/20F	8M/12F	10M/13F	35M/45F
MMSE	29.1 ± 0.9 SD	24.1 ± 4.7 SD	23.4 ± 4.2 SD	26.1 ± 4.3 SD

The mean age, breakdown by sex and Mini Mental State Examination (MMSE) scores are listed for each diagnostic group

All 80 study participants underwent MRI scanning on the 1.5‐Tesla Siemens Avanto scanner at the MRI Center at the University of California Los Angeles (UCLA). Standard anatomical T1‐weighted sequences were collected (256 × 256 matrix; voxel size = 1 × 1 × 1 mm^3^; TI = 900 ms; TR = 2,000 ms; TE = 2.89 ms; flip angle = 40°), and DWIs using single‐shot multisection spin‐echo echo‐planar pulse sequence (144 × 144 matrix; voxel size: 2 × 2 × 3 mm^3^; TR = 9,800 ms; TE = 97 ms; flip angle = 90°; scan time = 5 min 38 s). Thirty‐one separate images were acquired for each DWI series: one T2‐weighted image with no diffusion sensitization (*b*_0_ image) and 30 DWI volumes (*b* = 1,000 s/mm^2^).

To correct for head motion and eddy current distortions, all raw DWI volumes were aligned to the *b_0_* image using FSL (http://www.fmrib.ox.ac.uk/fsl). To ensure alignment in space, the skull‐stripped T1‐weighted images were linearly aligned to the standard Colin27 template (Holmes et al., 1998] using the FLIRT function in FSL with 9 degrees of freedom to account for translation, rotation and scaling in 3D [Jahanshad et al., 2012]. Then, we corrected for echo‐planar imaging (EPI)‐induced distortions, which can cause artifacts at fluid‐tissue interfaces and may lead to partial volume effects. We did this by linearly and elastically aligning the skull‐stripped *b*_0_ images to their corresponding T1‐weighted structural images using inverse consistent registration with a mutual information cost function [Leow et al., 2005]. The resulting deformation fields were applied to all 30 diffusion volumes before computing fractional anisotropy (FA) and mean diffusivity (MD) maps.

Using FreeSurfer version 5.3 (http://surfer.nmr.mgh.harvard.edu/) [Fischl, 2004], we automatically extracted 34 cortical labels [Desikan et al., 2006] per hemisphere from the T1‐weighted structural scans. All parcellations were closely inspected and edited as needed. Resulting cortical labels and T1‐weighted images were downsampled to the space of the linearly aligned DWIs using nearest neighbor interpolation to avoid intermixing of labels [Jahanshad et al., 2012]. Finally, to ensure that reconstructed fiber tracts would intersect labeled cortical boundaries, we dilated the labels with a 5 × 5 × 5 voxel isotropic box kernel. In this study, we did not include subcortical parcellations. We expect that the analysis of additional regions would redefine the organization of the network and the rich club might include subcortical labels. For now, in line with several other studies assessing networks, we chose to focus on cortical network connectivity, however, we intend to include the subcortical parcellations in our future work.

### *N* × *N* Connectivity Matrix Computation

Using the linearly aligned DWIs, we ran probabilistic tractography using a global approach based on the Hough transform [Aganj et al., 2011] and recovered ∼40,000 usable fibers (3‐D curves) for each participant. Tensor reconstruction was based on the diffusion tensor imaging (DTI) model. During processing, we filtered out fibers that were <20 mm in length, as they are more likely to be false positive fibers, and removed all duplicates. The 3‐D elastic deformation field obtained from the EPI distortion corrections were applied to the tracts' coordinates.

**Figure 1:**
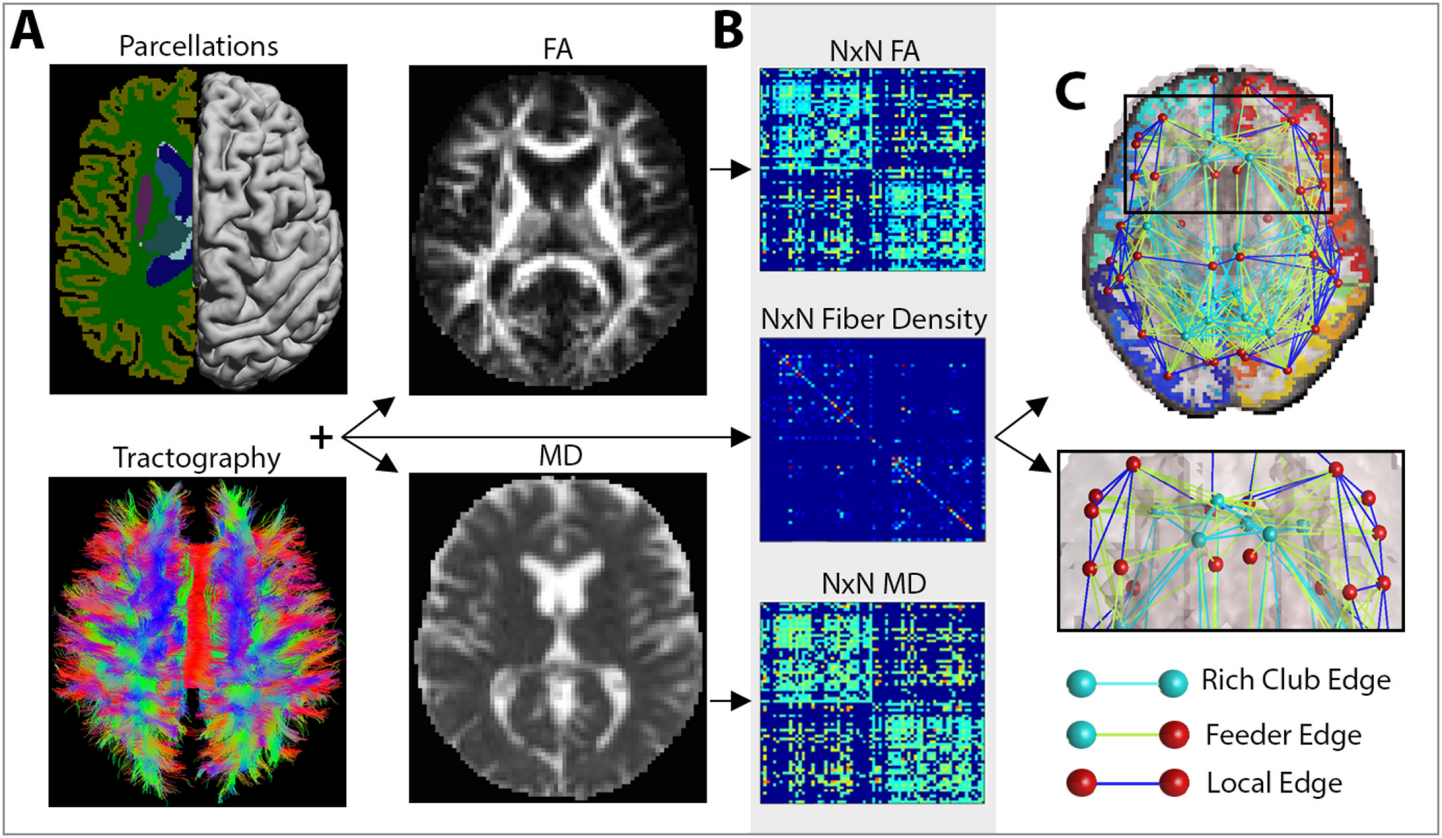
Image processing steps. (**A**) A subject's T1‐weighted anatomical image was segmented into *N* (i.e., 68) distinct ROIs, to define the network nodes in the connectome. These were combined with white matter tractography from the DWIs, fractional anisotropy (FA) and mean diffusivity (MD) maps to determine the number of detected fibers that interconnected a pair of nodes and the FA and MD values associated with them. (**B**) The interconnecting fibers were stored as *N* × *N* connectivity matrices describing the fiber density, average FA and average MD values among connections that linked pairs of nodes in the brain. (**C**) These connections can be represented as networks of nodes and edges that make up the human connectome. Based on the number and type of nodes that they pass through, edges can be defined as rich club edges that link rich club nodes to other rich club nodes, feeder edges, that link rich club nodes to nonrich club nodes and local edges that link nonrich club nodes to other nonrich club nodes. [Color figure can be viewed in the online issue, which is available at http://wileyonlinelibrary.com.]

Next, for each subject, we created three distinct connectivity matrices by combining fiber tractography with the downsampled cortical labels from FreeSurfer to map the brain's gross fiber connections (Fig. 1). These we enumerated in a 68 × 68 connectivity matrix with 34 regions of interest (ROIs) in each hemisphere. In this article, we use the word “fiber” to denote a single curve, or “streamline”, extracted via tractography; if no participants had detected fibers connecting two regions (i.e., all participants had a 0 count at a specific matrix element), then that connection was not included in the analysis. The three distinct connectivity matrices for each participant described (1) normalized fiber density of connections, (2) average FA, and (3) average MD along the tracts connecting a pair of ROIs. Finally, we used the connectivity matrices to define each participant's brain network—as a set of nodes (ROIs) and edges (fiber pathways).

### Computing the Weighted Rich Club Coefficient

The matrices allowed us to detect the rich club organization of a network—where high‐degree nodes are more interconnected among themselves than predicted by chance [Colizza et al., 2006]. The weighted rich club (Φ^w^) incorporates the weight of the edges within each network and is a function of nodal degree, *k*—defined as the sum of all the white matter connections that pass through an ROI. Φ^w^ is computed as the ratio between the sum of weights of a subnetwork of connections with nodal degree >*k*, $E_{>k}$, and the sum of strongest weights of top connections in the whole network [van den Heuvel and Sporns, 2011] (more details in Supporting Information). We normalized Φ^w^ using 500 random networks of the same size and similar nodal distribution to the networks from the participants in the study, to help determine if the observed connectivity densities exceeded those predicted by a random null distribution [Colizza et al., 2006]. A rich club phenomenon is said to be present if the normalized Φ^w^, or Φ_n_, is > 1 at distinct nodal levels, *k*. Using the Brain Connectivity Toolbox [Sporns, 2011], we computed the normalized weighted rich club coefficients as functions of the fiber density, FA‐ and MD‐weighted connectivity matrices. For simplicity, we will refer to these as Φ^w^_f_, Φ^w^_FA_, and Φ^w^_MD_.

### Network Nodes and Edges

We defined the rich club network at a previously reported nodal level of *k* > 15, separating the network into low‐ and high‐nodal degree rich club regimes [Daianu et al., 2013, 2015b). This rich club threshold has been previously proposed by studies using parcellation schemes of similar resolution [Daianu et al., 2014; van den Heuvel and Sporns, 2011; van den Heuvel et al., 2013]. To further explain, at *k*=16, each node in the subnetwork is required to connect to at least another 24% of the 67 nodes in the whole brain's network (16/67 = 24%, while at 100% one node connects to 67 nodes). We also ranked the most interconnected nodes at the aforementioned rich club threshold and selected the top 12% of the most consistently interconnected nodes across all diagnostic groups. This was in line with previous work by van den Heuvel et al. [2013]. The nodes that made up the top most interconnected subnetworks were included in the rich club network (see the Results section).

Based on the organization and interconnectedness of nodes in the network, edges (*E*) were defined as rich club, feeder, and local connections (Fig. 1C). Rich club connections linked rich club nodes to other rich club nodes, feeder connections linked rich club nodes to nonrich club nodes and local connections linked nonrich club nodes to other nonrich club nodes [Ball et al., 2014; van den Heuvel et al., 2013]. We illustrate these edges in the form of “connectograms” using software developed by Irimia and Van Horn [2014], Irimia et al. [2012], and Van Horn et al. [2012].

### Statistical Analyses

We performed two analyses to characterize brain connectivity at global and nodal level. For our global analyses, we tested for group differences (i.e., in Φ^w^_f_, Φ^w^_FA_, Φ^w^_MD_, *E*, rich club, feeder, and local connections) between patients and healthy controls and between the two patient groups. To do this, we ran a linear regression (coding disease status as 1 and controls as 0) on the residuals of the global measures after removing age, sex, and brain volume effects. Permutation testing was used to randomize the independent variable of interest (i.e., disease status) while maintaining the residuals of the global measures true to the subject. We performed *m* = 10,000 permutations and generated permutation‐corrected *P* values using the following formula: *P* = (*b*+1)/(*m*+1), where *b* is the number of randomized test statistics *t*_perm_ found to have a greater magnitude than the observed test statistic *t*_obs_. FDR correction [Benjamini and Hochberg, 1995] (*q* < 0.05) was applied to the *P* values to correct for multiple comparisons across all *k* values.

For our nodal analyses, we tested for diagnostic group differences in the connectivity matrices and separately, nodal degree, using the residual values as described above. To determine if the affected edges in the group differences were disproportionally distributed among rich club, feeder, or local edges, we performed a chi‐squared (*χ^2^*) test between the expected proportions of edges in each diagnostic group (i.e., known number of average rich club, feeder, and local edges) and the observed proportions (i.e., altered edges). Similarly, we tested for the distribution of altered rich club versus nonrich club nodes in each group. In the Results section, we will only report proportion of affected nodes and edges that were differently distributed than expected.

Finally, to determine if there were any changes in the nodal and global network measures with cognitive decline, we tested for associations between MMSE scores and each network measure (Φ^w^_f_, Φ^w^_FA_, Φ^w^_MD_, *E*, rich club, feeder, and local connections). For this, we ran a linear regression on the residual values as described above in all 80 participants and covaried for disease status. Permutation testing was also performed.

## RESULTS

### Global Analysis

**Figure 2:**
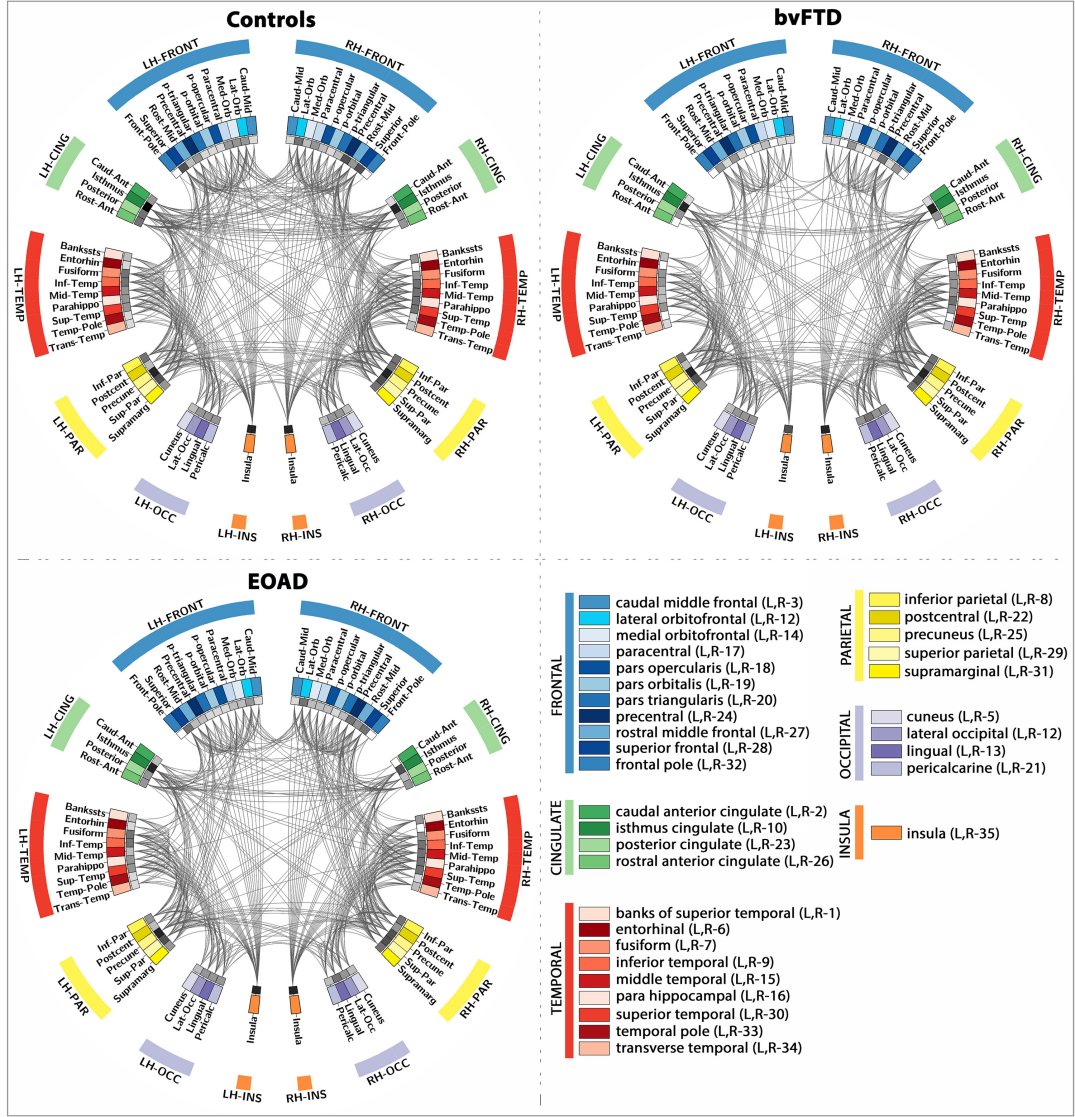
Connectograms depicting the average patterns of brain connectivity in healthy controls, bvFTD and EOAD patients. This parcellation scheme was based on the Desikan‐Killiany atlas provided with FreeSurfer, which is broken down into 34 cortical ROIs for the left and right hemispheres (LH and RH). Color coded ROIs are listed in the bottom right quadrant, and the nodal degree at each ROI is depicted by the inner ring (gray pallet, with darker squares indicating higher degree). [Color figure can be viewed in the online issue, which is available at http://wileyonlinelibrary.com.]

**Figure 3:**
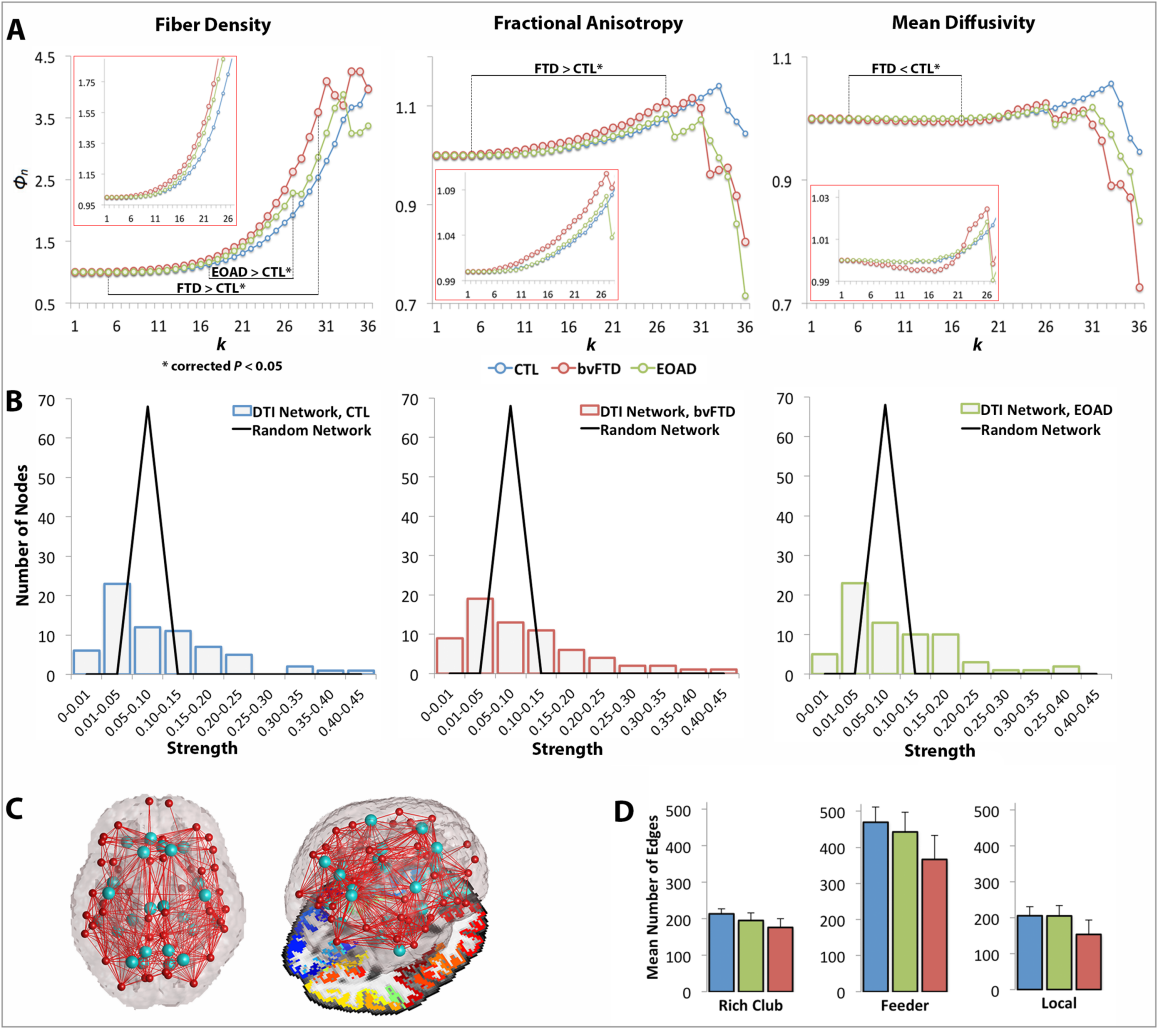
Rich club curves, strength distribution, rich club nodes and network edges. (**A**) Average normalized rich club curves (Φ_n_) computed as a function of fiber density (Φ^w^_f_), FA (Φ^w^_FA_) and MD connectivity (Φ^w^_MD_) in healthy controls (blue), bvFTD (red), and EOAD patients (green). The range of significantly (*) altered rich club measures is indicated (i.e., FTD < CTL*), as well as the zoomed in curves for *k* = 1–26 (in red boxes). bvFTD patients had a higher Φ^w^_f_ and Φ^w^_FA_ across the low and high *k*‐value regime and lower Φ^w^_MD_ curves in the low regime than seen in controls, reflecting lower levels of connectivity in the brain connectome; EOAD had a higher Φ^w^_f_ curve than controls in the high *k*‐value regime. (**B**) Fat‐tailed distribution of strength in the DTI networks of each diagnostic group and corresponding random networks indicating higher probability of hubs than in the random distribution. (**C**) Brain networks delineating 13 distinct regions (26 bilateral) included in the rich club network (light blue); figure to the right shows the 3D glass brain projected on top of an axial slice with FreeSurfer parcellations. (**D**) Average network connections and their standard deviation across the three diagnostic groups were defined as rich club, feeder, and local edges. All three classes of connections were lower in the diagnostic groups, relative to controls, except the local connections in EOAD patients. [Color figure can be viewed in the online issue, which is available at http://wileyonlinelibrary.com.]

A rich club organization was detected in the brain networks of patients and healthy controls. Patients had a significantly reduced connectivity (see fewer edges interconnecting ROIs in Fig. 2) and altered rich club organization (Fig. 3A), compared to controls:

### Global Analysis: bvFTD versus Controls

In bvFTD, the Φ^w^_f_ curve was higher than in healthy controls (FDR critical *P*_perm_ = 6 × 10^−4^, 10,000 permutations) at *k* = 5–30 (Fig. 3A), accompanied by a significant drop in *E* (*P*_perm_ = 10^−4^) in the whole brain networks, suggesting disrupted organization for the fiber density network. If in particular nodal degree, and therefore, fiber density, are lower in patients (see next section), then due to normalization (when the network components are equally distributed), the rich club density would go up as observed in Supporting Information, Eq. (S3), rather than down. To explain further, both the true and random Φ^w^ are < 1 as the *k*‐level increases; yet naturally, random Φ^w^ < true Φ^w^ especially when random Φ^w^ is constructed based on a lower degree diseased network, leading to larger ratios for the normalized Φ^w^. Supporting this, the Φ^w^_FA_ curve was also higher in bvFTD compared to controls (FDR critical *P*_perm_ = 7.5 × 10^−3^) at *k* = 5–27, while the Φ^w^_MD_ curve, expected to have an opposing effect (in neurodegeneration when FA decreases, MD increases), was lower in bvFTD, relative to controls (FDR critical *P*_perm_ = 0.015), at *k* = 5–17. Relative to healthy controls, bvFTD patients also had a significant drop in the number of edges among rich club (*P*_perm_ = 10^−4^), feeder (*P*_perm_ = 10^−4^) and local (*P*_perm_ = 10^−4^) connections in the network.

### Global Analysis: EOAD versus Controls

The Φ^w^_f_ curve increased in EOAD, relative to controls (FDR critical *P*_perm_ = 0.02), only in the high *k*‐value regime, at *k* = 17–27 (Fig. 3A). Φ^w^_FA_ and Φ^w^_MD_ were not significantly different between EOAD participants and controls, but the total *E* dropped significantly in EOAD, compared to controls (*P*_perm_ = 6 × 10^−4^). Rich club (*P*_perm_ = 10^−4^) and feeder (*P*_perm_ = 10^−4^) connections also decreased in EOAD, relative to controls. Local connections were not detectably different between EOAD and controls.

### Global Analysis: bvFTD versus EOAD

Between the two patient groups, bvFTD participants had a significantly different network than EOAD participants. bvFTD had a higher Φ^w^_f_ curve than EOAD (FDR critical *P*_perm_ = 0.03) at *k* = 4–30, and a significantly lower *E* (*P*_perm_ = 5 × 10^−4^). bvFTD also had a higher Φ^w^_FA_ curve at *k* = 5–27 (FDR critical *P*_perm_ = 0.025) and lower Φ^w^_MD_ curve than EOAD at *k* = 5, 6, 8, 10, 11, 13–18 (FDR critical *P*_perm_ = 0.021). In addition, bvFTD patients had a significant lower number of edges in the feeder (*P*_perm_ = 1.7 × 10^−3^) and local (*P*_perm_ = 10^−4^) connections, than EOAD.

### Node and Edge Analysis

The fiber density in our DTI networks had a fat‐tailed distribution, following an exponentially truncated power law [Clauset et al., 2009; Crossley et al., 2014]. This indicates the presence of strongly interconnected hub nodes (Fig. 3B). The most interconnected nodes of the rich club network were, as previously documented in Sporns et al. [2007], the right and left hemisphere precuneus, posterior cingulate, superior parietal, superior frontal, and insula (on average, their nodal degree ≥37 out of 68 possible regions in healthy controls and most patients). Although these constitute highly connected epicenters in the connectome, they only add up to 6% of the connectome. To be consistent with previous work that selected the top 12% most consistently interconnected nodes in each brain network [van den Heuvel et al., 2013], we included additional nodes with equivalently high average nodal degree (≥30): the right and left isthmus of the cingulate, fusiform, inferior temporal, lateral orbitofrontal, lingual, parahippocampal, precentral and rostral anterior cingulate. The only exceptions to these were the right fusiform and medial orbitofrontal (nodal degree < 30) but we included them in the rich club for consistency. Overall, we analyzed 26 rich club bilateral nodes and 42 nonrich club nodes (Fig. 3C).

### Nodal Analysis: bvFTD versus Controls

**Figure 4:**
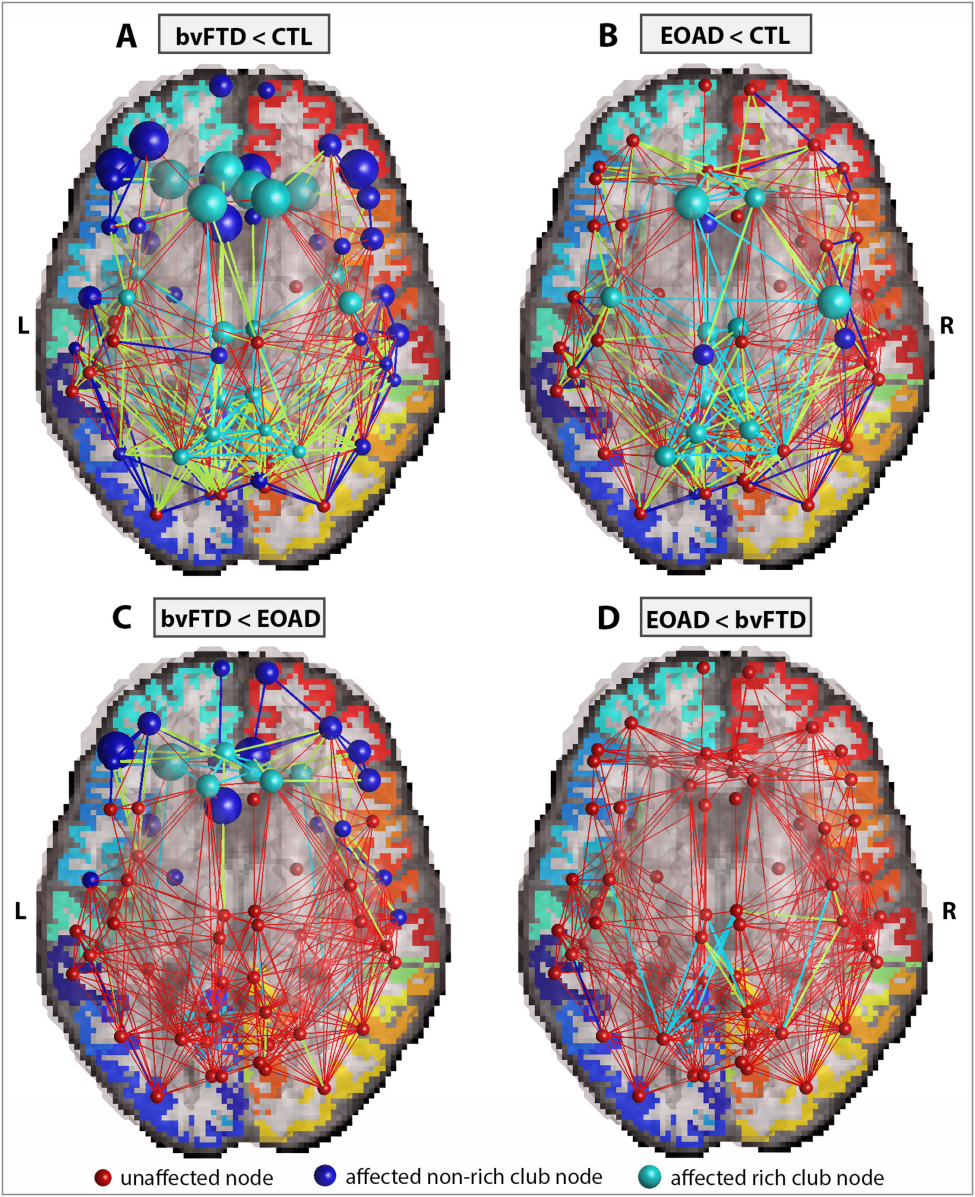
Average networks indicating nodal degree differences between diagnostic groups interconnected by altered rich club (light blue), feeder (light green) and local connections (dark blue). (**A**) Decreases in nodal degree in bvFTD, versus controls, at 53 distinct nodes proportionally distributed among rich club (light blue) and nonrich club (dark blue) nodes; note that most significantly altered nodes were in the frontal lobe. (**B**) Decreases in nodal degree in EOAD, versus controls, across 19 nodes with largest proportion of these nodes located in the rich club network. (**C**) Decreases in nodal degree in bvFTD, versus EOAD, at 24 proportionally distributed rich club and nonrich club nodes. (**D**) Only the lingual area in EOAD presented with a decreased nodal degree, compared to bvFTD. All affected edges from group comparisons assessing decreases in fiber density, FA and MD connectivity matrices (also shown in Fig. 5) were added onto the average networks in bvFTD (A, C) and EOAD (B, D). These show existing connections between significantly altered nodes that may allow the disease propagation between regions of the brain. The size of the nodes is proportional to the magnitude of the *P*_obs_ (the greater the sphere, the more significant *P*_obs_). [Color figure can be viewed in the online issue, which is available at http://wileyonlinelibrary.com.]

**Figure 5:**
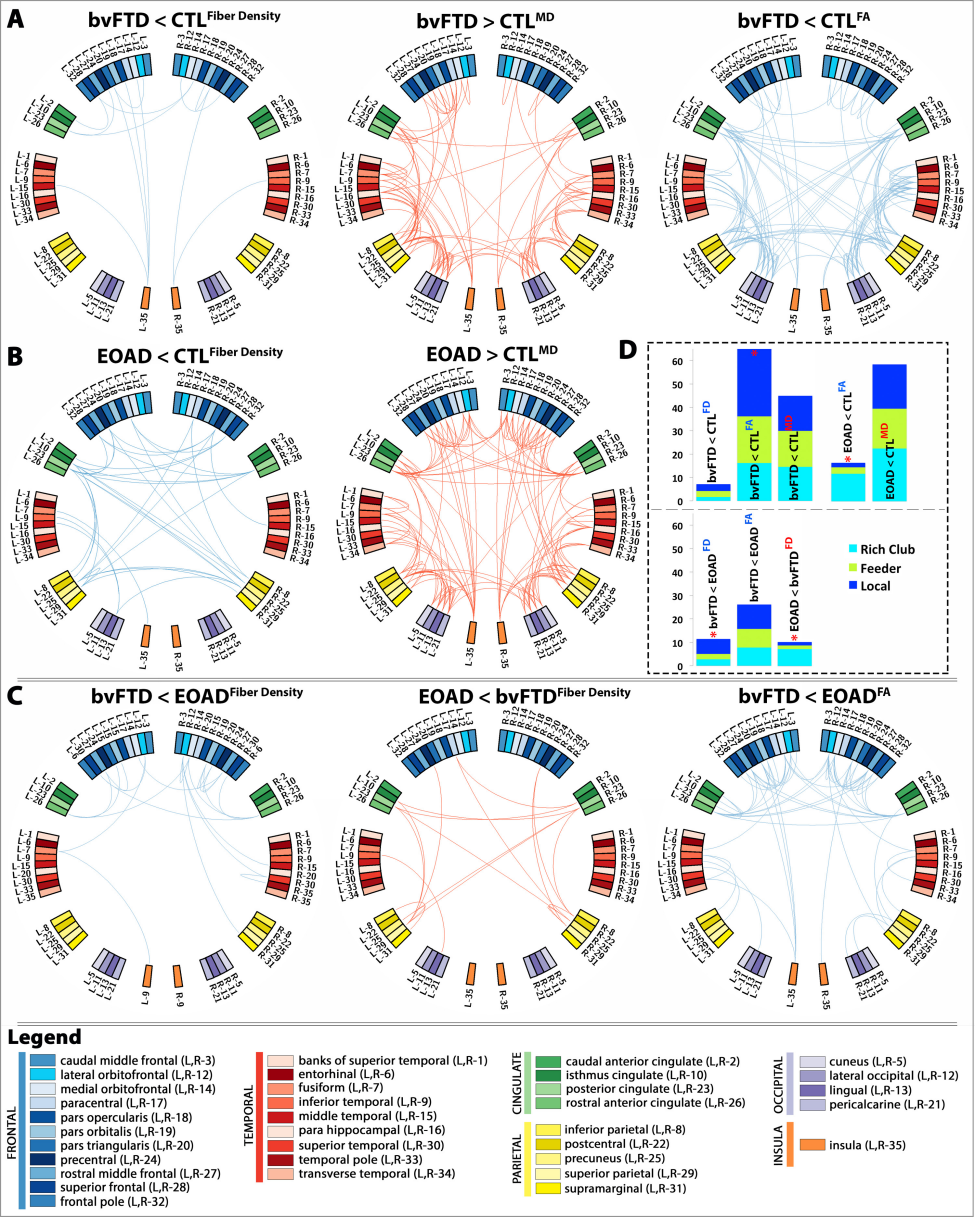
Diagnostic group differences in the *N* × *N* connectivity matrices for fiber density (FD), FA and MD between: (**A**) bvFTD and healthy controls; (**B**) EOAD and healthy controls; and (**C**) bvFTD and EOAD. Blue edges indicate decreasing patterns for corresponding measures, while red edges indicate increasing patterns—all suggestive of alterations in connectivity. (**D**) Proportion (%, *y*‐axis) of significantly altered connections (100% × observed/expected) illustrated as rich club, feeder, or local edges. In bvFTD, the proportion of local edges was more affected among the altered connections when compared to controls and separately, EOAD; while in EOAD, the proportion of rich club edges was more affected among the altered connections when compared to controls and bvFTD. “<” indicates that a particular diagnostic group presented with more alterations than the other; “*” denotes where a significantly higher proportion of edges than expected was altered as described by the *χ* ^2^ test. [Color figure can be viewed in the online issue, which is available at http://wileyonlinelibrary.com.]

The nodal degree was significantly lower in bvFTD patients, relative to controls in 53 regions (FDR critical *P*_perm_ = 0.03; Fig. 4A). Some of the most affected areas were predominantly in the frontal lobe, as well as temporal and parietal (Table S1, Supporting Information). Twenty‐one of the affected nodes (21/26 = 80%) were part of the rich club network and 32 (32/42 = 76%) were in the nonrich club (Table 2). The fiber density was lower in bvFTD, than controls (FDR critical *P*_perm_ = 5.5 × 10^−3^), at 18 distinct connections in the connecome. Furthermore, FA connectivity analysis detected decreased FA (FDR critical *P*_perm_ = 0.020) across 158 connections; the proportion of affected connections was differently distributed than expected among the three edge categories in bvFTD: 29 were rich club connections out of a total average of 179 (Fig. 3D), 78/390 feeder and 51/173 local connections (χ^2^ P = 6.5 × 10^−3^; Fig. 5D). In particular, the proportion of local connections was more altered than the rich club connections in bvFTD (χ^2^ P = 3.0 × 10^−3^), and than the feeder connections (χ^2^ P = 0.013). Meanwhile, MD connectivity increased at 112 connections (FDR critical *P*_perm_ = 0.028; Fig. 5A).

**Table 2:** Group differences for bvFTD versus controls, EOAD versus controls and bvFTD versus EOAD for assessing changes in nodal degree, fiber density, FA and MD connectivity.

Nodal degree	Fiber Density Connectivity	FA Connectivity	MD Connectivity
**bvFTD < Controls**			
**FDR crt. *P_perm_*=0.03** 53/68 affected nodes NS (χ^2^ *P* > 0.05)	**FDR crt. *P_perm_*=5.5 × 10^−3^** 18/742 affected edges NS (χ^2^ *P* > 0.05)	**FDR crt. *P_perm_*=0.020** 158/742 affected edges local < RC (χ^2^ *P=*3.0 × 10^−3^) local < feeder (χ^2^ *P=*0.013)	**FDR crt. *P_perm_*=0.028** 112/742 affected edges NS (χ^2^ *P* > 0.05)

**EOAD < Controls**			
**FDR crt. *P_perm_*=0.012** 19/68 affected nodes RC < non‐RC (χ^2^ *P=*1.4 × 10^−3^)	**FDR crt. *P_perm_*=4.2 × 10^−3^** 38/843 affected edges RC < feeder *(*χ^2^ *P=*3.6 × 10^−6^) RC < local (χ^2^ *P=*8.3 × 10^−5^)	NS (*P_perm_* > 0.05)	**FDR crt. *P_perm_*=0.015** 158/843 affected edges NS (χ^2^ *P* > 0.05)

**bvFTD < EOAD**			
**FDR crt. *P_perm_*=5.7 × 10^−3^** 24/68 affected nodes NS (χ^2^ *P* > 0.05)	**FDR crt. *P_perm_*=4.2 × 10^−3^** 25/742 affected edges local < RC (χ^2^ *P=*0.016)	**FDR crt. *P_perm_*=5.8 × 10^−3^** 63/742 affected edges NS (χ^2^ *P* > 0.05)	NS (*P_perm_* > 0.05)

**EOAD < bvFTD**			
**FDR crt. *P_perm_*=5.7 × 10^−3^** 1/68 affected nodes NS (χ^2^ *P* > 0.05)	**FDR crt. *P_perm_*=4.2 × 10^−3^** 25/843 affected edges RC < feeder (χ^2^ *P*=2.6 × 10^−4^) RC < local (χ^2^ *P*=4.5 × 10^−3^)	NS (*P_perm_* > 0.05)	NS (*P_perm_* > 0.05)

We determined the proportion of affected (observed) rich club (RC) versus nonrich club nodes among the significant findings, in addition to the distribution of altered edges among rich club, feeder and local edges in the brain networks; these are listed as part of the existing (expected) nodes and edges averaged across each diagnostic group (i.e., 158/742 edges in bvFTD). Results are illustrated in **Figure 5**. Overall, among the altered connections the proportion of local edges in bvFTD (when compared to controls and EOAD) were more affected than their rich club and feeder connections; whereas for EOAD, the proportion of rich club nodes and edges were more affected than their nonrich club nodes, feeder or local edges. NS=not significant; ‘<’=more altered node/connection; crt. =critical.

### Nodal Analysis: EOAD versus Controls

Nodal degree also decreased in EOAD, relative to controls (FDR critical *P*_perm_ = 0.012) at 19 ROIs (Fig. 4B). Some of the most significant deficits were found in the left and right hemisphere precentral regions, left lingual, left and right precuneus, and posterior cingulate among other regions (Table S2, Supporting Information). Thirteen of the affected nodes in EOAD were in the rich club (13/26 = 50%) and 6 were nonrich club network (6/42 = 14%). The proportion of rich club nodes was more severely affected than the nonrich club nodes (χ^2^ P = 1.4 × 10^−3^). Fiber density was lower in EOAD, versus controls (FDR critical *P*_perm_ = 4.2 × 10^−3^) at 38 different connections. Twenty‐three affected connections out of a total of 195 average EOAD edges were among rich club connections, 12/442 were feeder, and 4/206 local (Fig. 5B). The proportion of affected connections was differently distributed than expected (χ^2^ P = 3.5 × 10^−7^), with rich club connections more severely affected than both feeder (χ^2^ P = 3.6 × 10^−6^) and local edges (χ^2^ P = 8.3 × 10^−5^) (Fig. 5D). MD connectivity was higher across 158 distinct connections in EOAD, relative to controls (FDR critical *P*_perm_ = 0.015). There were no differences detected in FA connectivity.

### Nodal Analysis: bvFTD versus EOAD

bvFTD patients had a lower nodal degree, relative to EOAD in 24 areas of the brain (FDR critical *P*_perm_ = 5.7 × 10^−3^; Fig. 4C and Table 2) mostly in the frontal lobe (Table S1, Supporting Information); only the left lingual presented with lower nodal degree in EOAD patients, relative to bvFTD. Fiber density decreased in bvFTD, relative to EOAD (FDR critical *P*_perm_ = 4.2 × 10^−3^), at 25 connections (5/179 rich club, 9/390 feeder, 11/173 local; Fig. 5C) and the proportion of connections among the three categories of edges was significantly different (χ^2^ P = 0.04), with local connections more severely affected than rich club edges (χ^2^ P = 0.016), but not feeder (χ^2^ P = 0.1); fiber density also decreased in EOAD, relative to bvFTD (FDR critical *P*_perm_ = 4.2 × 10^−3^), at 24 connections differently distributed among rich club, feeder, and local connections than expected (14/195 rich club, 7/442 feeder, 3/206 local; χ^2^ P = 1.4 × 10^−4^). As seen in EOAD versus controls, the proportion of rich club edges in EOAD was more affected than the feeder (χ^2^ P = 2.6 × 10^−4^) and local connections (χ^2^ P = 4.5 × 10^−3^). FA connectivity decreased in bvFTD participants, relative to EOAD at 63 distinct connections (FDR critical *P*_perm_ = 5.8 × 10^−3^). MD connectivity did not detect significant differences.

### Changes in Connectivity with Cognitive Decline

Among the global measures, MMSE scores decreased with the number of edges for the rich club connections (*P*_perm_ = 0.04). At local network level, MMSE scores decreased with increasing MD connectivity at 115 connections (FDR critical *P*_perm_ = 6.6 × 10^−3^) (29/196 rich club, 58/436 feeder, and 28/196 local). Here, the affected connections were tested against the average number of connections within each diagnostic group and were proportionally distributed among the rich club, feeder, and local connections. The affected connections traversed all lobes of the brain, especially the parietal lobe (inferior and superior segments, and precuneus) and the temporal lobe (inferior, middle and superior segments) bilaterally with preferential involvement of the left hemisphere. Furthermore, no other significant associations were detected between the remaining global and nodal network measures and cognitive decline. This may be because a larger sample is needed to pick up associations or MMSE scores are less sensitive than other more specific cognitive tests to changes in connectivity in the elderly.

## DISCUSSION

As hypothesized, we found disrupted levels of connectivity in patients with dementia, bvFTD, and EOAD (Fig. 2), based on an in‐depth analysis of the weighted rich club organization of the brain. In addition to the commonly studied fiber density metric, we also analyzed connectome changes in terms of standard diffusion tensor imaging (DTI) measures FA and MD—expected to decrease and increase simultaneously, indicating structural disruptions in the white matter.

### bvFTD and EOAD Affect the Global Network Organization of the Connectome

From our global analyses, we found that the fiber density and FA rich club curves were affected across both low and high degree nodes in bvFTD, versus controls, while MD only detected differences in the low nodal degree regime—possibly indicating that bvFTD predominantly affects the peripheral (local) nodes of the network (Fig. 3A). The low *k*‐value regime includes low degree nodes that form locally clustered and segregated communities of the connectome and provide a communication relay that aids the global integration of information in the brain [de Reus and van den Heuvel, 2013; Sporns, 2011]. Meanwhile, in EOAD, only the fiber density rich club curves detected alterations in the high *k*‐value regime targeting the rich club network nodes (Fig. 3A). The high *k*‐value regime retains only the most densely interconnected nodes of the connectome and defines the rich club that further reflects the integration of hubs in the network. Communication among these central nodes is achieved through long distance pathways and is crucial for efficient global information transfer in the healthy brain [van den Heuvel et al., 2009]. Although we show that the weighted rich club network is affected in both types of dementia, further investigation is needed to distinguish whether the rich club networks are more affected purely due to their high topological value (i.e., high nodal degree) or because there are disease processes that originate from or propagate to centrally located nodes.

### Nodal Analysis Reveals a 78% Altered Network in bvFTD and 28% in EOAD

In addition to studying the global rich club organization, we also analyzed the integrity of the connections that transfer information between hub and nonhub regions of the brain. The brain exhibits densely interconnected networks in which communication hubs and nonhubs operate collectively, rather than as individual entities [Daianu et al., 2015b; van den Heuvel and Sporns, 2011]. In our nodal analyses, we showed that in bvFTD, relative to healthy participants (Figs. 4A and 5A) and separately, EOAD (Figs. 4C and 5C), most prominent reductions were detected within proportionally distributed rich club and nonrich club nodes across the cortex. Predominantly, the local connections that linked these regions were most affected among all connections in the network. Conversely, in the EOAD versus controls (Figs. 4B and 5B), and separately bvFTD (Figs. 4D and 5C), disconnections were most obvious among the rich club nodes of the connectome and the rich club connections that linked them (Figs. 4B and 5B, D). Although rich club nodes were affected in EOAD, their nodal degree did not decrease below the rich club nodal degree requirement (≥30), unlike seen in bvFTD—where the right and left lateral and medial orbitofrontal regions (rich club regions in controls and EOAD) dropped out of the rich club subnetwork. This might indicate that the global rich club communication backbone in EOAD remains relatively preserved [Daianu et al., 2015a,b,c] despite the nodal differences detected by the weighted analyses.

These findings are in line with our knowledge of the pathology of bvFTD and EOAD. Patterns of anatomical changes do vary in bvFTD, and relate to the behavioral profile of the patients, but traditionally, bvFTD is associated with largely symmetrical atrophy of the frontal lobes [Whitwell et al., 2009]. Recent studies divide bvFTD into separate anatomical groups—frontal‐dominant, temporal‐dominant, frontotemporal, and frontotemporalparietal [Whitwell et al., 2009]. In this study, we found the disruption in connectivity to be more consistent with the frontotemporalparietal pathology in bvFTD. Ninety‐six percent of the bilateral frontal lobe nodes presented with a decreased nodal degree, compared to controls, and 70% of these were nonrich club nodes—possibly explaining why most alterations were located among the local connections in bvFTD (Fig. 4A). Ninety‐three percent of the biparietal nodes and 72% of the temporal nodes had a lower nodal degree in bvFTD, compared to controls. The nodes in the frontal, parietal, and temporal lobes were fairly symmetrically affected across the two hemispheres. The occipital lobe in bvFTD was relatively spared; only the right hemisphere cuneus had a lower nodal degree than seen in controls. The insula, especially in the right hemisphere, also contributed to the disconnection observed in bvFTD.

Moreover, the burden of pathology in EOAD has been associated with biparietal and frontal lobe dysfunction [Marshall et al., 2007] and left greater than right hemispheric temporal lobe atrophy [Koedam et al., 2010]. Unlike LOAD, EOAD often presents with prominent visual dysfunction [Koedam et al., 2010]. Here, we found considerable supporting evidence for a disrupted connectome in EOAD, versus controls, in areas relevant to disease that were predominantly located among the rich club network nodes. The nodes with a lower nodal degree that drove this phenomenon were located in the biparietal lobe (57% of parietal lobe was affected; Fig. 4B); some of these alterations were in the precuneus, posterior cingulate, and isthmus of the cingulate—regions known to show profound atrophy in AD [Daianu et al., 2015a; Thompson et al., 2003]. Twenty‐seven percent of the frontal lobe nodes had a lower nodal degree, including the superior frontal, caudal anterior, and middle cingulate affected predominantly in the left hemisphere. The inferior temporal and fusiform were affected in the left temporal lobe—areas of the brain that are responsible for identifying objects and face perception [Gross, 1994]. The nodes on the pericalcarine cortex (anatomical location of primary visual cortex [Bedny, 2011]) and lingual areas presented with a lower nodal degree in the left hemisphere occipital lobe. This is indicative of the visual impairment that is prevalent in EOAD [Koedam et al., 2010].

Many diseases may affect the brain in a network‐related spatial pattern that might closely relate to functional intrinsic connectivity networks [Buckner et al., 2005; Zhou et al., 2012]. Although this work did not assess functional connectivity, our structural findings are in line with reports on an atrophied “salience network” (SN) in bvFTD and an atrophied posterior “default mode network” (DMN) in AD [Zhou et al., 2010]. bvFTD patients presented with affected structural regions that overlap with areas that are part of the SN and include the anterior cingulate cortex and frontoinsular connections. One of the most affected nodes in the bvFTD connectome was the right hemisphere rostral anterior cingulate (Fig. 4A), also a rich club node, which plays a major role in the SN and is involved in emotion and decision‐making. This node may also be associated with a high level of nodal stress and could affect, possibly through transneuronal spread, its neighboring nodes including the right lateral orbitofrontal—another severely affected rich club node that is part of the SN and is concerned with decision‐making [Kringelbach, 2005]. The right and left superior frontal, part of the DMN and involved in self‐awareness [Goldberg et al., 2006], were greatly affected in both bvFTD and EOAD. The superior frontal is one of the most central rich club nodes in the connectome providing a communication relay for many neighboring nodes—which might possibly make it more vulnerable to disease processes. In addition, the precuneus and posterior cingulate, hubs in the DMN, were also affected in EOAD. The precuneus controls visuospatial functioning [Karas et al., 2007] while the posterior cingulate has a central role in the DMN, possibly in supporting internally directed cognition [Leech and Sharp, 2014]. Overall, these nodes that are actively involved in the SN and DMN, constitute network‐specific hubs in bvFTD and EOAD, which may or may not be sites of initial injury, but are closely related to the clinical deficits defining each form of dementia.

Another important aspect involves the impact of gray matter atrophy on network analysis. Atrophy is commonly reported in patients with bvFTD, especially in the frontal lobe as found by a recent meta‐analysis from 11 voxel‐based analysis studies across 237 bvFTD patients and 297 healthy controls [Pan et al., 2012]. Widespread gray matter changes were also found in EOAD patients in the parietal and occipital lobes, as well as in areas of the neocortex [Frisoni et al., 2007]. Gray matter loss can lead to secondary changes in the white matter structure, for example, through Wallerian degeneration [Thompson et al., 1998], as suggested by the altered connectivity in this study. Although the gray matter effects on the white matter structure are not well understood, lesions in the gray matter, as seen in traumatic brain injury, can affect the integration and segregation properties of brain networks [Irimia and Van Horn, 2014]. Particularly, lesions in the frontopolar and superior frontal cortex may lead to alterations in segregation properties [Irimia and Van Horn, 2014]—a local property of the network, and can be associated with changes in behavior and personality, as seen in bvFTD. This is further supported by the prominent local disruption among components of the bvFTD network shown in this study and could imply that gray matter deficits may contribute to these alterations. Frontal lobe lesions may only minimally affect integration properties, but injury to the medial parietal and superior temporal cortices may cause drastic alterations in network‐wide integration [Irimia and Van Horn, 2014]—an important characteristic of the connectome that gives it the capacity to engage in global interactions. The parietal lobe was found to be most altered in our EOAD patients and is also one of the regions with greatest connectivity in humans [Hagmann, 2008]. It, therefore, has major involvement in the rich‐club network. The brain may also be more sensitive to injuries in regions with more basic primary sensory functions—critical to the survival of the individual, and more resilient to injuries among the higher order functions (i.e., frontal pole regions) [Irimia and Van Horn, 2014].

### Limitations and Concluding Remarks

A limitation of this study is the DTI reconstruction that was used to recover fibers in the white matter structure. Unlike high angular resolution imaging (HARDI), DTI is not always able to accurately reconstruct the axonal branching, including crossing fibers, within a single image voxel [Daianu et al., 2015c]. This might lead to an underrepresentation of the white matter fiber populations in the human connectome [van den Heuvel and Sporns, 2011] and might result in false‐positive results, and false negatives. In our nodal analyses, we found unexpected increases in fiber density and FA connectivity in the dementia groups, compared to controls, primarily projecting through the insula. Although the microstructural aspects of abnormal increases in connectivity are not fully understood, there may be microscopic deficits at axonal level, as well as decreases in packing density and branching or decreases in axonal diameter [Beaulieu, 2002; Hoeft et al., 2007] that might lead to such results. Connectivity measures of fiber density, FA and MD are indirect markers of white matter integrity, yet they provide valuable information about the altered connectivity patterns in disease. Importantly, FA and MD metrics were sensitive to a large range of alterations and led to fewer abnormal increases (than found for fiber density), possibly suggesting more reliable outcomes in comparing healthy to diseased groups.

Another limitation of our study may be the low resolution parcellation scheme used to segment the brain into 68 cortical regions. Including more regional components may help better distinguish between functionally heterogeneous regions and possibly—better understand nodes that participate in multiple networks. However, the organizational principles of the structure and function of the connectome might be independent of the parcellation paradigm [de Reus and van den Heuvel, 2013] although the quantification of graph theory measures could change between low‐ to large‐resolution networks. Anatomical templates segment the brain into 50–120 regions, whereas many significantly larger parcellations schemes are designed to produce hundreds or thousands of parcels of approximately equal size [de Reus and van den Heuvel, 2013]. Nonetheless, it remains open for debate if rather uniformly sized brain regions have an advantage over anatomically delineated templates.

In this study, we report on a severely disrupted connectome in bvFTD affecting 78% of the network nodes, including both rich club and peripheral (local) components. Distinctly, the local connections that linked these components were most severely affected in bvFTD—unlike what we found in EOAD, where 28% of the connectome nodes were affected and predominated among the rich club nodes and their rich club connections. Longitudinal studies are needed to characterize the transneuronal degeneration across the network‐like organization of the brain, but an important step is understanding the elements of the network, as shown here, that may enable dysfunction to spread between linked regions of the connectome.

## Supporting information

Supporting Information
