# Supplementary material for: Disrupted rich club network in behavioral variant frontotemporal dementia and early‐onset Alzheimer's disease
Source: Hum Brain Mapp. 2015 Dec 17;37(3):868–83. doi: 10.1002/hbm.23069 (PMC4883024; doi:10.1002/hbm.23069)
Supplement: Supplementary file 1 — Supporting Information [file HBM-37-868-s001.docx]

**Supplementary Information**

**METHODS**

**Computation of the Weighted Rich Club Coefficient**

The *weighted rich club coefficient* is a function of the nodal degree, *k* – the number of edges that connect to a node. At a particular *k* level the nodal degree is computed as:

$k=\sum_{j\in N} a_{ij}$ (**Eq. S1**)

where *k* is the degree of a node *i*, and *a_ij_* is a connections status between nodes *i* and *j* (*a_ij_* =1 if nodes *i* and *j* are connected and *a_ij_* =0 otherwise) (Watts & Strogatz, 1998) (Daianu et al., 2013).

We computed the rich club coefficient for each subject’s anatomical network at a range of *k* value thresholds (*i.e.,* *k*=1-30). To do this, we examined subnetworks, ***M***, in the connectivity matrix, and computed the nodal degree by counting the links that interconnected each node *i* in the subnetwork with *k* other nodes. Nodes that had a nodal degree ≤ *k* were removed from the network. Then, we ranked all the connections in the network as a function of weight and stored them in a vector, ***W^ranked^***. Within ***M***, we selected the degrees larger than *k*; the number of links between the components of the subnetwork was counted, ***E_>k_*,** as well as the sum of their collective weight, ***W_>k_***. Then, the weighted rich club, *Φ^w^(k)*, was computed as the ratio between ***W_>k_*** and the sum of the ranked weights from ***W^ranked^*** (from the whole network) given by the top strongest connections in ***E_>k_*** (van den Heuvel & Sporns, 2011).

(**Eq. 2**)

To normalize the measures, we compared the observed values to a rich club coefficient computed on an average calculated from 500 randomized networks of equal size and similar connectivity distribution.

 (**Eq. 3**)

| **Group Comparisons** | **Affected Areas of the Brain with Lower Nodal Degree** |
| --- | --- |
| **bvFTD vs. controls**  (FDR critical *P_perm_*=0.03) | **Frontal lobe:**  rostral anterior cingulate (left 1.26x10^-12^ and right *p_obs_*=7.7x10^-15^)  caudal middle frontal (*p_obs_*=5.6x10^-4^ and 1.7x10^-4^)  *pars opercularis* (*p_obs_*=7.8x10^-3^ and 1.2x10^-5^)  *pars orbitalis* (*p_obs_*=7.9x10^-12^ and 5.2x10^-12^)  *pars triangularis* (*p_obs_*=6.2x10^-^7 and 1.1x10^-9^)  lateral orbitofrontal (*p_obs_*=1.9x10^-13^ and 2.3x10^-4^)  medial orbitofrontal (*p_obs_*=1.6x10^-10^ and 8.3x10^-15^)  precentral (*p_obs_*=8.3x10^-4^ and 1.4x10^-9^)  rostral middle frontal (*p_obs_*=1.2x10^-11^ and 2.0x10^-9^)  caudal anterior cingulate (*p_obs_*=4.3x10^-3^ and 9.6x10^-11^)  frontal pole (*p_obs_*=3.1x10^-5^ and 0.028)  superior frontal (*p_obs_*=7.7x10^-17^ and 1.5x10^-12^)  left paracentral (*p_obs_*=1.5x10^-4^)  **Temporal lobe:**  entorhinal (left *p_obs_*=2.3x10^-4^)  temporal pole (*p_obs_*=1.5x10^-7^ and 6.6x10^-6^)  middle temporal (*p_obs_*=0.029 and 7.1x10^-7^)  superior temporal (*p_obs_*=1.2x10^-5^ and 5.0x10^-5^)  Inferior temporal (*p_obs_*=1.6x10^-7^ and 8.5x10^-6^)  **Parietal lobe:**  inferior parietal (*p_obs_*=9.8x10^-3^ and 1.4x10^-3^)  isthmus of the cingulate (*p_obs_*=8.3x10^-5^ and 1.5x10^-3^)  posterior cingulate (*p_obs_*=1.0x10^-9^ and 6.1x10^-4^)  precuneus (*p_obs_*=1.2x10^-3^ and 0.027)  right supra-marginal (*p_obs_*= 0.028)  **Ocicpital lobe:**  right cuneus (*p_obs_*= 3.3x10^-3^)  **Other**:  insula (*p_obs_*=1.7x10^-6^ and 4.0x10^-4^) |
| **EOAD vs. controls**  (FDR critical *P_perm_*=0.012) | **Frontal lobe:**  superior frontal areas (*p_obs_*=2.8x10^-5^ and 8.0x10^-4^)  caudal anterior cingulate (left *p_obs_*=3.2x10^-3^)  caudal middle frontal (left *p_obs_*=0.012)  paracentral (left *p_obs_*=5.1x10^-3^)  precentral (*p_obs_*=2.8x10^-4^ and *p_obs_*=3.2x10^-7^)  **Temporal lobe:**  inferior temporal (left *p_obs_*=8.7x10^-3^)  fusiform (left *p_obs_*=1.1x10^-4^)  **Parietal lobe:**  precuneus (*p_obs_*=5.1x10^-3^ and 7.0x10^-3^)  superior parietal (left *p_obs_*=1.2x10^-4^)  peri-calcarine (left *p_obs_*=9.5x10^-3^)  isthmus of the cingulate (left *p_obs_*=6.4x10^-3^ and 4.2x10^-3^)  posterior cingulate (*p_obs_*=5.5x10^-4^ and 2.0x10^-3^)  superior parietal (left *p_obs_*=1.2x10^-4^)  postcentral (right *p_obs_*=4.9x10^-4^)  **Occipital lobe:**  lingual (left *p_obs_*=1.9x10^-5^)  **Other:**  insula (right *p_obs_*=9.4x10^-3^) |
| **bvFTD vs. EOAD**  (FDR critical *P_perm_*=5.7x10^-3^) | **Frontal lobe:**  right and left hemisphere superior frontal (*p_obs_*=9.2x10^-5^ and 1.2x10^-5^)  *pars orbitalis* (*p_obs_*=5.8x10^-7^ and 1.4x10^-8^)  *pars triangularis* (*p_obs_*=5.8x10^-6^ and 3.4x10^-3^)  lateral orbitofrontal (*p_obs_*=2.8x10^-8^ and 2.1x10^-8^)  medial orbitofrontal (*p_obs_*=7.1x10^-9^ and 2.5x10^-5^)  frontal pole (*p_obs_*=2.9x10^-5^ and 4.8x10^-3^) |
| **EOAD vs. bvFTD**  (FDR critical *P_perm_*=5.7x10^-3^) | **Ocicpital lobe:**  lingual (left *p_obs_*=4.2x10^-3^) |

**Table S1.** Group differences for bvFTD vs. controls, EOAD vs. controls and bvFTD vs. EOAD for assessing changes in nodal degree in the brain network. Areas of the brain (nodes) where altered nodal degree metrics were detected are listed for each hemisphere (left first, then right), as applicable. FDR corrected *p*-values are included, as well as the *p_obs_* at each significantly altered node.

**Additional References:**

Daianu, M., Jahanshad, N., Nir, T. M., Toga, A. W., Jack, C. R., Jr., Weiner, M. W., . . . Alzheimer's Disease Neuroimaging, I. (2013). Breakdown of brain connectivity between normal aging and Alzheimer's disease: a structural k-core network analysis. *Brain Connect, 3*(4), 407-422. doi: 10.1089/brain.2012.0137

van den Heuvel, M. P., & Sporns, O. (2011). Rich-club organization of the human connectome. *J Neurosci, 31*(44), 15775-15786. doi: 10.1523/JNEUROSCI.3539-11.2011

Watts, D. J., & Strogatz, S. H. (1998). Collective dynamics of ‘small-world’ networks. *Nature, 393*(4), 440-442.
